# Supplementary material for: Cadmium in the Soil–Tea–Infusion Continuum of Selenium-Enriched Gardens: Implications for Food Safety
Source: Foods. 2025 Sep 10;14(18):3156. doi: 10.3390/foods14183156 (PMC12470203; doi:10.3390/foods14183156)
Supplement: Supplementary file 1 [file foods-14-03156-s001.zip › foods-3816586-supplementary.docx]

# Table S1 Basic properties of sampling sites in Se-enriched tea gardens

| Sampling point | locations | Tea cultivar | Soil type | Tea tree ages (a) | Elevation (m) | Fertilizers |
| --- | --- | --- | --- | --- | --- | --- |
| no. 1 | Xianyu Mountain | Zhu ye | Humic Umbrisols | 100 | 720 | Urea and organic fertilizer, along with superphosphate and potassium chloride were applied every two years. |
| no. 2 | Xianyu Mountain | Zhu ye | Humic Umbrisols | 100 | 620 | Urea and organic fertilizer, along with superphosphate and potassium chloride were applied every two years. |
| no. 3 | Xianyu Mountain | Zhu ye | Humic Umbrisols | 100 | 575 | Urea and organic fertilizer, along with superphosphate and potassium chloride were applied every two years. |
| no. 4 | Shuang keng village | Liu ye | Humic Umbrisols | 100 | 630 | Urea and organic fertilizer, along with superphosphate and potassium chloride were applied every year. |
| no. 5 | Cha yuan li village | Liu ye | Dystric Cambisols | 100 | 425 | Urea and organic fertilizer, along with superphosphate and potassium chloride were applied every year. |
| no. 6 | Cha yuan li village | Zhu ye | Dystric Cambisols | 70 | 425 | Urea and organic fertilizer, along with superphosphate and potassium chloride were applied every year. |
| no. 7 | Cha yuan li village | Zhu ye | Dystric Cambisols | 70 | 375 | Urea and organic fertilizer, along with superphosphate and potassium chloride were applied every year. |
| no. 8 | Hong jia village | Zhu ye variety,Jiu han | Dystric Cambisols | 70 | 400 | Urea and organic fertilizer, along with superphosphate and potassium chloride were applied every year. |
| no. 9 | Hong jia village | Zhu ye variety,Jiu han | Dystric Cambisols | 70 | 430 | Urea and organic fertilizer, along with superphosphate and potassium chloride were applied every year. |
| no. 10 | Hong jia village | Zhu ye variety,Jiu han | Dystric Cambisols | 70 | 370 | Urea and organic fertilizer, along with superphosphate and potassium chloride were applied every year. |
| no. 11 | Li jia village | Zhu ye variety,Jiu han | mountainous Haplic acrisols | 70 | 350 | Urea and organic fertilizer, along with superphosphate and potassium chloride were applied every year. |
| no. 12 | Li jia village | Zhu ye variety,Jiu han | mountainous Haplic acrisols | 70 | 415 | Urea and organic fertilizer, along with superphosphate and potassium chloride were applied every year. |

**Table** S**2 The operating conditions and usage parameters of ICP-MS**

| Instrumental Conditions | Set value | Operational Parameters | Set value |
| --- | --- | --- | --- |
| Carrier gas flow rate | 14 mL/min | RF power | 1200 W |
| Auxiliary gas flow rate | 0.8 mL/min | Atomizer pressure | 0.9～1.0 bar |
| Sampling depth | 5 mm | Plasma gas flow rate | 13.0 |
| Plasma power | 1550 W | Scanning mode | Peak Shaving |
| Helium gas flow in the collision cell | 4.8mL/min | Each mass number measurement point | 3 |
| Atomizer flow rate | 1 L /min | Integral time | 10 ms |
| Pulsator pump speed | 40 r/min | Interface temperature | 30.67℃ |
| Data acquisition mode | KED | Measurement method | Pulse/Analog |
| Number of replicates | 3 | Pulsator pump speed | Analysis,20 rpm; rinse:70 rpm |

**Table** S**3** **Properties of Se-enrich tea garden soil**

| Sampling site | pH | SOM  (g kg^−1^) | TN  (mg kg^−1^) | TP  (%) | AP  (mg kg^−1^) | AK  (mg kg^−1^) | C/N |
| --- | --- | --- | --- | --- | --- | --- | --- |
| no. 1 | 5.81±0.24^cde^ | 85.33±12.14^a^ | 4.36±0.58^ab^ | 0.17±0.05^a^ | 3.13±0.81^abc^ | 117.67±10.53^bcd^ | 11.35±0.60^a^ |
| no. 2 | 5.75±0.10^cde^ | 35.53±2.96^b^ | 2.63±0.77^bc^ | 0.06±0.04^bc^ | 3.27±1.94^bc^ | 142.00±19.8^ab^ | 8.70±3.82^a^ |
| no. 3 | 5.58±0.19^e^ | 35.04±19.76^b^ | 5.27±2.93^a^ | 0.11±0.03^abc^ | 1.40±0.46^abc^ | 113.67±16.76^bcde^ | 6.13±5.04^a^ |
| no. 4 | 6.14±0.08^cd^ | 28.13±2.39^b^ | 1.61±0.21^c^ | 0.04±0.00^c^ | 7.03±6.41^abc^ | 126.33±25.04^bc^ | 10.39±2.48^a^ |
| no. 5 | 5.86±0.15^cde^ | 42.63±4.76^b^ | 2.05±0.55^c^ | 0.08±0.02^bc^ | 5.20±2.36^ab^ | 105.00±21.97^cdef^ | 12.55±2.21^a^ |
| no. 6 | 6.80±0.34^a^ | 34.83±10.25^b^ | 2.15±0.46^c^ | 0.12±0.03^ab^ | 3.20±0.60^abc^ | 96.67±13.1^cdef^ | 9.68±4.12^a^ |
| no. 7 | 6.72±0.42^ab^ | 31.03±10.09^b^ | 1.78±0.15^c^ | 0.11±0.01^abc^ | 5.30±1.75^c^ | 72.00±8.83^f^ | 10.09±3.84^a^ |
| no. 8 | 6.70±0.19^ab^ | 29.53±8.26^b^ | 2.07±0.32^c^ | 0.11±0.06^abc^ | 3.40±1.73^abc^ | 79.00±15.58^ef^ | 8.79±4.11^a^ |
| no. 9 | 5.64±0.13^de^ | 36.37±1.03^b^ | 2.58±0.66^bc^ | 0.08±0.02^bc^ | 4.77±1.30^abc^ | 170.33±18.26^a^ | 8.64±2.28^a^ |
| no. 10 | 5.67±0.21^de^ | 44.67±6.5^b^ | 2.21±0.39^bc^ | 0.10±0.01^bc^ | 6.80±1.31^abc^ | 142.67±15.17^ab^ | 12.14±3.80^a^ |
| no. 11 | 6.23±0.22^bc^ | 27.43±6.82^b^ | 2.29±0.24^bc^ | 0.10±0.03^abc^ | 2.47±0.98^a^ | 90.00±11.31^cdef^ | 7.01±2.44^a^ |
| no. 12 | 5.72±0.23^cde^ | 43.6±4.95^b^ | 2.72±0.52^bc^ | 0.12±0.03^ab^ | 4.27±1.03^abc^ | 86.67±3.77^def^ | 9.48±1.27^a^ |
| Mean | 6.05±0.49 | 39.51±17.40 | 2.64±1.42 | 0.10±0.03 | 4.19±1.72 | 111.83±32.50 | 9.58±3.27 |

Note: SOM, soil organic matter; TN, total nitrogen; TP, total phosphorus; AP, available phosphorus; AK, Available Potassium; C/N, carbon nitrogen ratio. Different superscript letters within the same column indicate statistically significant differences (*p* < 0.05).
